# Selective Infiltration Etching as a surface modification for high-translucency zirconia: Comparative analysis of roughness, composition, and bond strength

**DOI:** 10.12688/f1000research.176295.1

**Published:** 2026-04-18

**Authors:** Bushra Alakeel, Ibrahim Alghoraibi, issam jamous

**Affiliations:** 1 Department of Fixed Prosthodontics, Faculty of Dental Medicine, Damascus University, Damascus, Damascus Governorate, 011, Syria; 2 Department of Physics, Faculty of Science, Damascus University, Damascus, Damascus Governorate, 011, Syria

**Keywords:** Zirconia, Roughness, Morphology, Resin, Cement, Bonding, selective Infiltration Etching (SIE), shear bond strength (SBS)

## Abstract

**Background:**

Zirconia is widely regarded as a highly promising material for dental prosthetics due to its excellent biocompatibility and mechanical strength, making it an attractive alternative to metal restorations. However, its chemical inertness and high hardness pose challenges for bonding with resin cements, potentially leading to clinical debonding.

**Methods:**

Thirty-six high-translucency zirconia cubes (10 × 10 × 10 mm) were randomly assigned to three groups: Group 1 – sandblasting; Group 2 – CoJet system; Group 3 – SIE. Surface roughness, morphology, and composition were assessed using a profilometer, SEM/EDX, and AFM. Following bonding with resin cement, all specimens underwent 5,000 thermocycles. Shear bond strength was then measured.

**Results:**

The SIE group demonstrated significantly greater surface roughness and bond strength compared with the other groups. SEM analysis revealed that SIE generated a well-defined nanoporous surface promoting deep resin infiltration. In contrast, sandblasting and CoJet treatments produced irregular, less retentive surface morphologies.

**Conclusion:**

These findings indicate that SIE provides a superior micromechanical and chemical interface, thereby enhancing the durability of zirconia–resin bonding.

## Introduction

Ceramic systems have been developed to eliminate metal substructures and enhance light reflection, resulting in restorations that meet the aesthetic demands of dental patients with exceptional quality. ^1, 2^ Zirconia ceramics, particularly yttria-stabilised zirconia, are widely used in clinical applications due to their high toughness and fracture resistance, making them especially suitable for implant abutments and frameworks in dental restorations. ^3, 4^ However, despite their excellent mechanical properties, zirconia-based dental ceramics exhibit limited bond strength to resin cements. ^5^

This limitation arises from zirconia’s high crystalline content and the absence of a glassy phase, which renders its surface resistant to conventional acid etching. As a result, effective micromechanical interlocking is hindered, thereby reducing the bonding efficacy of methacrylate-based resin cements. ^3, 6^ To address this challenge, various mechanical and chemical surface modification techniques have been proposed to improve the adhesion between resin cement and zirconia. ^7^

Mechanical methods such as aluminum oxide air abrasion and the tribochemical silica coating technique have been explored to increase surface roughness and improve micromechanical retention. ^8^ While aluminum oxide air abrasion remains the most commonly used approach, it can introduce surface defects that compromise the structural integrity of ceramics. ^9^ In contrast, the tribochemical silica coating offers an alternative, enabling salinisation or the use of resin cements that contain functional monomers, such as 10-methacryloyloxydecyl dihydrogen phosphate (MDP). ^10^

Recently, there has been a growing interest in modern approaches such as the selective infiltration etching (SIE) technique. This relatively new innovation has demonstrated promising results in enhancing the adhesive potential of zirconia surfaces. The SIE method incorporates a heat-induced maturation (HIM) process, wherein controlled short thermal cycles induce stress at zirconia grain boundaries. A low-fusing glass infiltrates these regions, forming a three-dimensional porous network that is subsequently etched with hydrofluoric acid to create a roughened surface conducive to resin bonding. ^8^

Despite advancements in the field, a universally accepted “gold standard” for bonding zirconia has not yet been established. Moreover, the combined effects of sandblasting and selective infiltration etching (SIE) on highly translucent zirconia, as well as their long-term influence on adhesive bond durability, remain insufficiently explored. ^11^ To address this knowledge gap, the present study evaluated three surface treatment methods—Selective Infiltration Etching (SIE), CoJet, and conventional sandblasting—to enhance zirconia-resin adhesion.

The null hypothesis of this study is that Selective Infiltration Etching (SIE) does not significantly affect surface roughness, morphology, and composition when compared to traditional methods.

## Materials and methods

### Study design

This comparative in vitro experimental study was conducted at the Department of Fixed Prosthodontics, Faculty of Dental Medicine, Damascus University, between October 2024 and March 2025. Ethical approval was obtained from the Ethics Committee at Damascus University (Approval No. 2023/3664).

The sample size was determined using G*Power 3.1.9, based on the study by Kwon *et al*. ^12^ (Effect size f = 1.575, α error probability = 0.05, power (1 − β error probability) = 0.95, number of groups = 3). Although thirty zirconium cubes were initially required, the study was conducted on thirty-six zirconium cubes to enhance accuracy.

### Samples preparation

Thirty-six highly translucent cubic zirconia blocks (10 × 10 × 10 mm ^3^) were used in this study. The specimens were ultrasonically cleaned in distilled water for 3 minutes, then sequentially polished with silicon carbide papers to achieve a smooth, glossy surface. All specimens were randomly allocated into three equal groups (n = 12), each subjected to a distinct surface treatment protocol.

### Surface treatment protocols

Group 1 (Air-abrasion), specimens were surface-treated by blasting with 50 μm aluminum oxide particles articles at a pressure of 2.5 bar for 20 seconds, from a distance of 10 mm.

Group 2 (CoJet System) specimens were treated with 30 μm silica-coated alumina particles (CoJet, 3 M ESPE) under a pressure of 2.8 bar.

Group 3 (selective infiltration etching, SIE) specimens were coated with a laboratory-prepared paste consisting of 65% silica, 15% alumina, 10% sodium oxide, 5% potassium oxide, and 5% titanium oxide, mixed with distilled water. The coated specimens underwent thermal infiltration at 750 °C for 3 minutes, followed by cooling and etching with 9.5% hydrofluoric acid for 20 minutes.

The Ra value, measured in micrometres (μm), was used as the surface roughness indicator in this study, before and after surface treatment with a profilometer (TR200, Time Group, USA). Measurements were conducted at the Faculty of Mechanical and Electrical Engineering, University of Damascus. The stylus scanned a cutoff length of 0.8 mm three consecutive times, and the average Ra value was recorded for each specimen.

All zirconia blocks were bonded to standardized composite resin cylinders using a dual-cure MDP-containing resin cement (Theracem BISCO, USA) according to the manufacturer’s instructions. Cementation was performed under a constant load using a standardized metal device. Subsequently, all specimens were subjected to 5000 thermocycles between 5 °C and 55 °C with a dwell time of 30 seconds.

### Microstructural analysis

To further evaluate of surface morphology, atomic force microscopy (AFM) measurements were performed in tapping mode under ambient conditions (26–28 °C). Silicon cantilevers (Tap190 Al-G, NanoSensors™, Neuchâtel, Switzerland) with 30-nm-thick aluminum reflex coating were used. According to the manufacturer’s datasheet, the cantilever spring constant ranged from 1.5–15 N/m, and the tip radius was less than 10 nm. The scan rate was set at 1 Hz, and the scanning size was 5 × 5 μm ^2^. Three-dimensional images were reconstructed using Nanosurf Digital Surf software (NanoSurf ^®^, Liestal, Switzerland), and the area roughness parameter (Sa) was recorded to assess the average surface roughness.

Two specimens from each group were examined using scanning electron microscopy (SEM) (VEGA II, XMMU, TESCAN, Czech Republic). SEM analysis was performed on the treated zirconia surfaces at accelerating voltage of 30 kV and a scan speed of 6–7 ms/pixel. Images were acquired using both backscattered electron (BSE) and secondary electron (SE) detectors at various magnifications to evaluate surface morphology. The same specimens were subsequently analyzed using energy-dispersive X-ray spectroscopy (EDX) to assess elemental composition.

Shear Bond Strength (SBS) testing was performed using a universal testing machine at a crosshead speed of 1 mm/min until failure occurred. The maximum load (N) at failure was recorded and divided by the bonding area to calculate bond strength in megapascals (MPa).

Failure modes were examined under SEM and categorized as adhesive failure (at the zirconia–cement interface), cohesive failure (within the resin cement), or mixed failure (combination of adhesive and cohesive failures).

### Statistical analysis

Statistical analysis was conducted using SPSS software (version 21.0; IBM Corp., Armonk, NY, USA). Data were first assessed for normality using the Kolmogorov–Smirnov test. As the results indicated a normal distribution (p > 0.05), parametric tests were applied. Mean and standard deviation values were calculated for all variables. One-way analysis of variance (ANOVA) was used to compare mean shear bond strength (SBS) across groups. Tukey’s post hoc test was employed to evaluate pairwise differences in surface roughness and SBS values. A significance level of p < 0.05 was considered statistically significant.

## Results

### 1- Morphologic results

**Figure 1.**
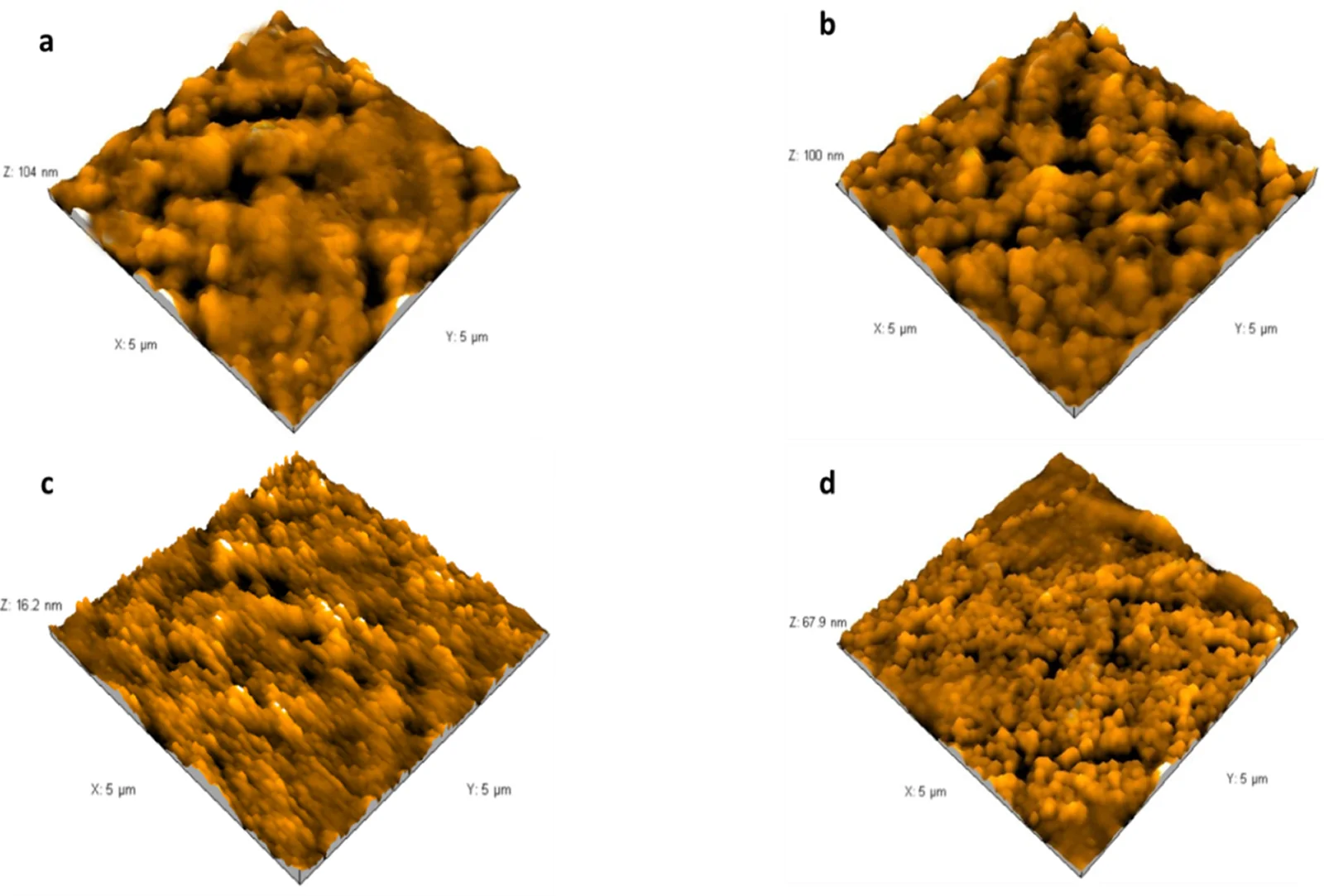
Typical three-dimensional images of the zirconia surface after treatments. (a) sandblasted group, (b) Cojet, (c) SIE and (d) SIE after etching.

AFM was used to evaluate surface morphology, revealing distinct topographical variations among the treated zirconia surfaces. The sandblasted group exhibited irregular granular elevations interspersed with large, compact clusters. In contrast, the CoJet group showed nodular agglomerates with uneven spatial distribution. The SIE group demonstrated well-defined, sub-parallel ripple-like grooves and the greatest height variation, indicative of increased surface roughness. Following etching, the SIE specimens presented a uniformly textured surface characterized by dense, fine spherical granules and numerous micro- to nano-scale pores. Quantitative analysis confirmed that the SIE group exhibited the highest roughness parameters (Ra and Sa), aligning with profilometer measurements and underscoring the method’s effectiveness in producing a highly retentive surface suitable for durable resin bonding ( Figure 1).

Following the surface treatments, the SEM examination showed that sandblasted specimen exhibited an irregular topography characterized by deep grooves and a pitted morphology with sharp edges and angular peaks resulting from the impact of alumina particles. This roughened surface increased the available area for micromechanical interlocking; however, it also revealed localized microcracks and stress concentration zones that may compromise structural integrity. In contrast, CoJet-treated surfaces displayed isolated clusters of silica-rich nodules and “islands” adhered to the zirconia substrate.

**Figure 2.**
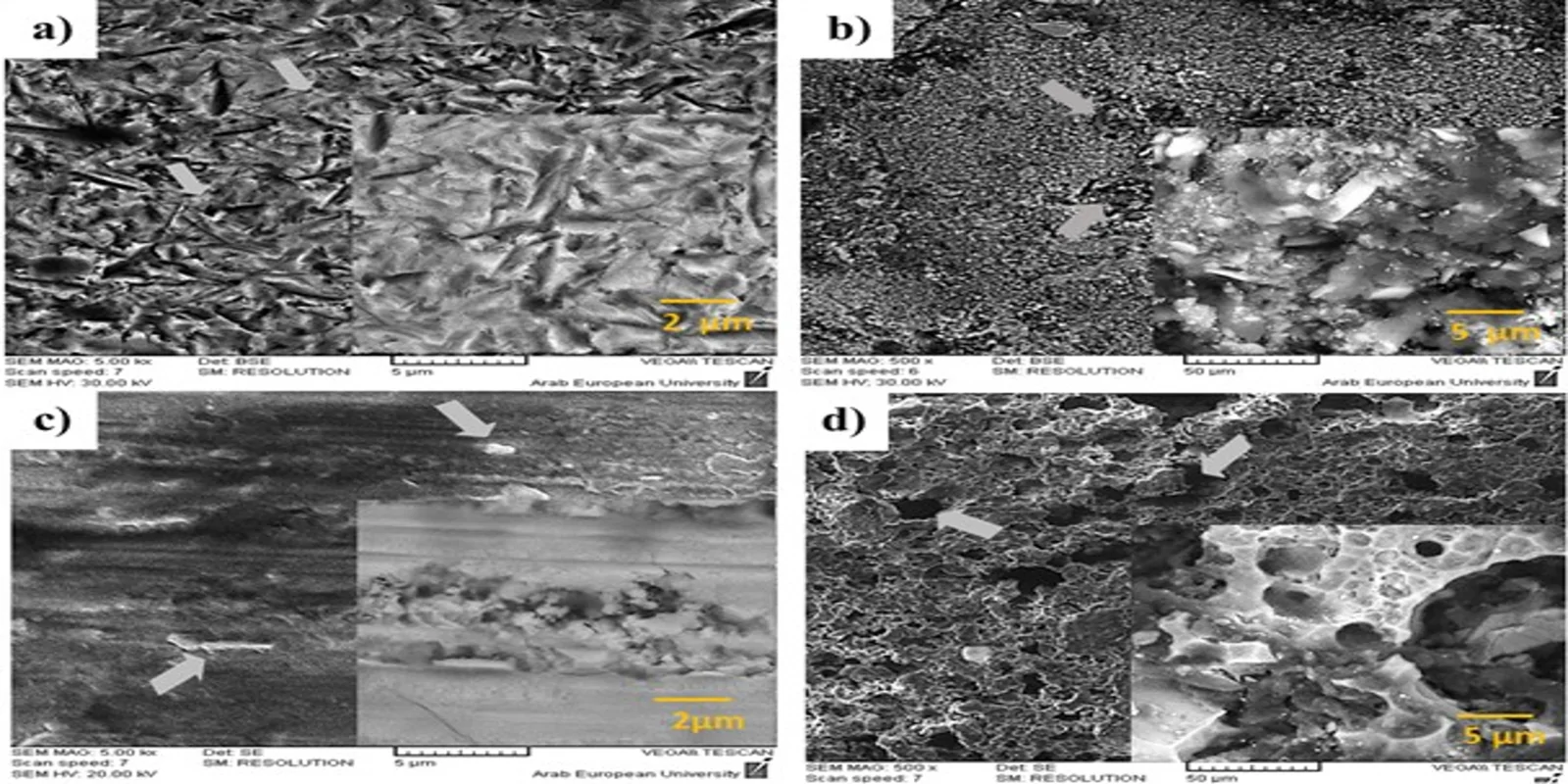
SEM images of zirconia surface after treatments (a) sandblasted, (b) CoJet, (c) SIE and (d) SIE after etching. The grey arrow indicates sharp edges in group a, while it points to clusters of silica islands in group b. fused round beads were observed in group c, and group d appeared honeycomb –like due to the etching.

These alumina deposits, coated with silica, imparted moderate surface reactivity and roughness, potentially enhancing silane coupling. Although their distribution across the surface was non-uniform. The specimens treated with SIE before etching exhibited a dense, glazed-like surface composed of molten, spherical glassy beads formed through thermal infiltration of the silica-alumina-alkali glass layer into grain boundaries. This process yielded a chemically modified, compact surface free from mechanical defects. Following hydrofluoric acid etching, the SIE-treated specimens developed a distinct honeycomb-like micro/nanoporous structure resulting from the selective dissolution of the infiltrated glassy phase. This topography featured an interconnected network of pores and channels, enhancing micromechanical retention and resin infiltration, thereby promoting stronger adhesion between the resin cement and zirconia ( Figure 2).

**Figure 3.**
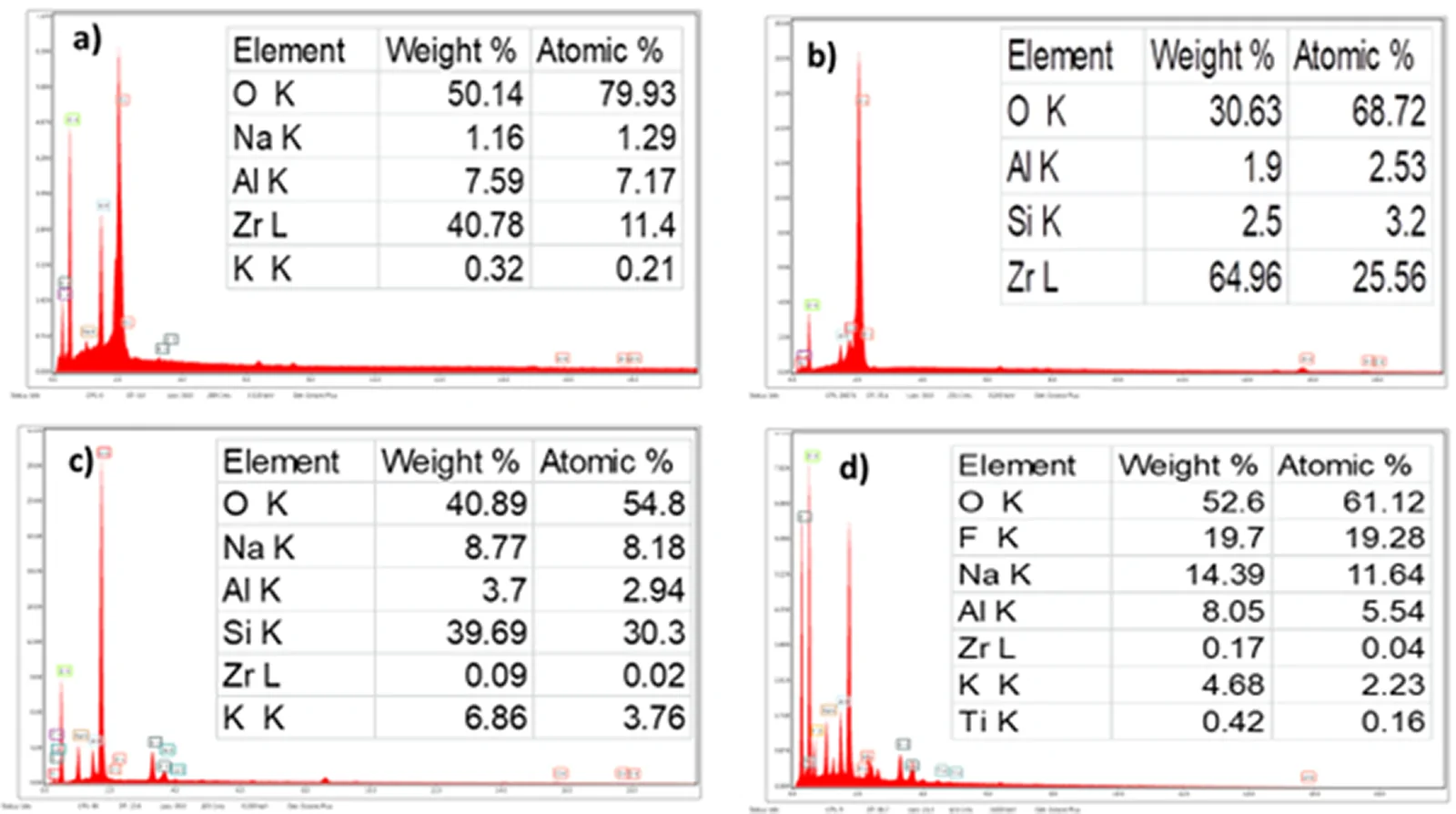
EDX analysis showed considerable compositional variations in the treated zirconia surfaces.

EDX analysis revealed pronounced compositional variations across the treated zirconia surfaces. In the sandblasted group, oxygen (50.14 wt%) and zirconium (40.78 wt%) were the predominant elements, accompanied by detectable traces of aluminum (7.59 wt%) originating from alumina abrasion particles. In the CoJet group, silica (Si: 2.5 wt%) was identified, and the aluminum signal decreased, indicating the deposition of SiO _2_-coated alumina particles onto the zirconia surface. The SIE-treated samples exhibited a markedly different elemental profile characterized by elevated levels of silicon (39.69 wt%), sodium (8.77 wt%), and potassium (6.86 wt%), while zirconium content was significantly reduced (0.09 wt%). This suggests that the zirconia surface was entirely covered by a glassy infiltration layer. Following HF etching (group d), the glassy phase partially dissolved, resulting in a surface enriched with oxygen (52.6 wt%), fluorine (19.7 wt%), and sodium (14.39 wt%), while trace amounts of zirconium (0.17 wt%) remained Compared to the previous group, zirconium content decreased significantly, while silicon, sodium, and potassium increased after SIE treatment but declined following HF etching. The presence of fluorine in this group confirms a chemical interaction between HF and the glass phase. Collectively, these findings indicate that SIE treatment effectively modifies zirconia surface chemistry through glass infiltration and subsequent etching, producing a compositionally active surface that enhances the durability of resin bonding ( Figure 3).

**Figure 4.**
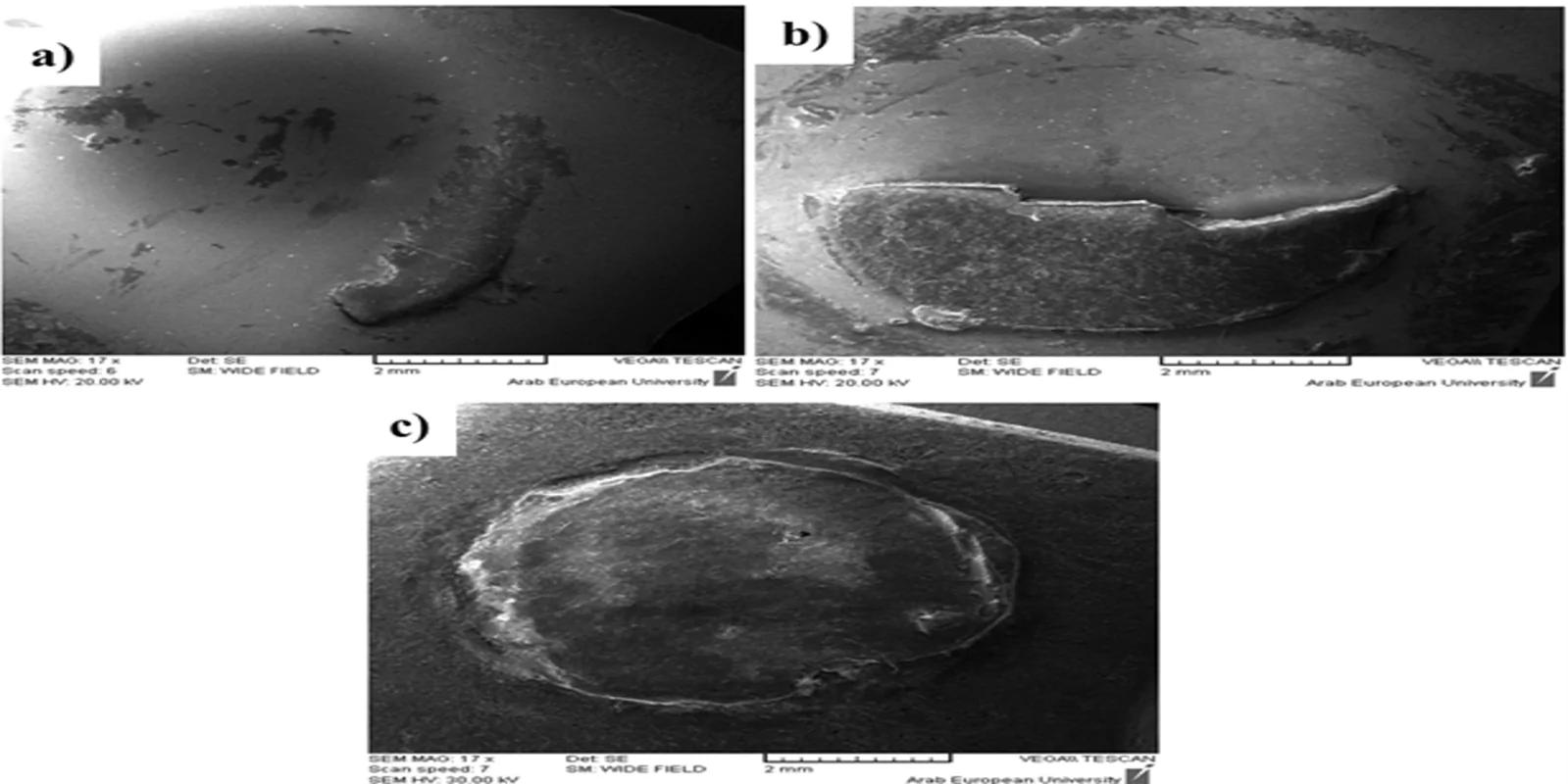
SEM images of failure types at a magnification of 17×. (a) Adhesive failure, (b) mixed failure, and (c) cohesive failure.

SEM analysis revealed distinct failure modes among the experimental groups. In the CoJet and sandblasted groups, predominantly adhesive failures were observed, characterized by clean zirconia surfaces with minimal traces of resin. This result suggests weak interfacial bonding. In contrast, the specimens treated with SIE primarily exhibited cohesive failures, with fractures occurring within the resin cement and the zirconia surfaces fully covered by resin residues. This observation indicates a strong micromechanical and chemical bond. Additionally, some mixed failures were observed, marked by partial resin residues exhibiting intermediate adhesion strength between the two extremes. These findings align with the quantitative data, confirming that the SIE surface treatment achieved the most reliable and strongest bond between resin and when zirconia compared to conventional methods ( Figure 4).

**Figure 5.**
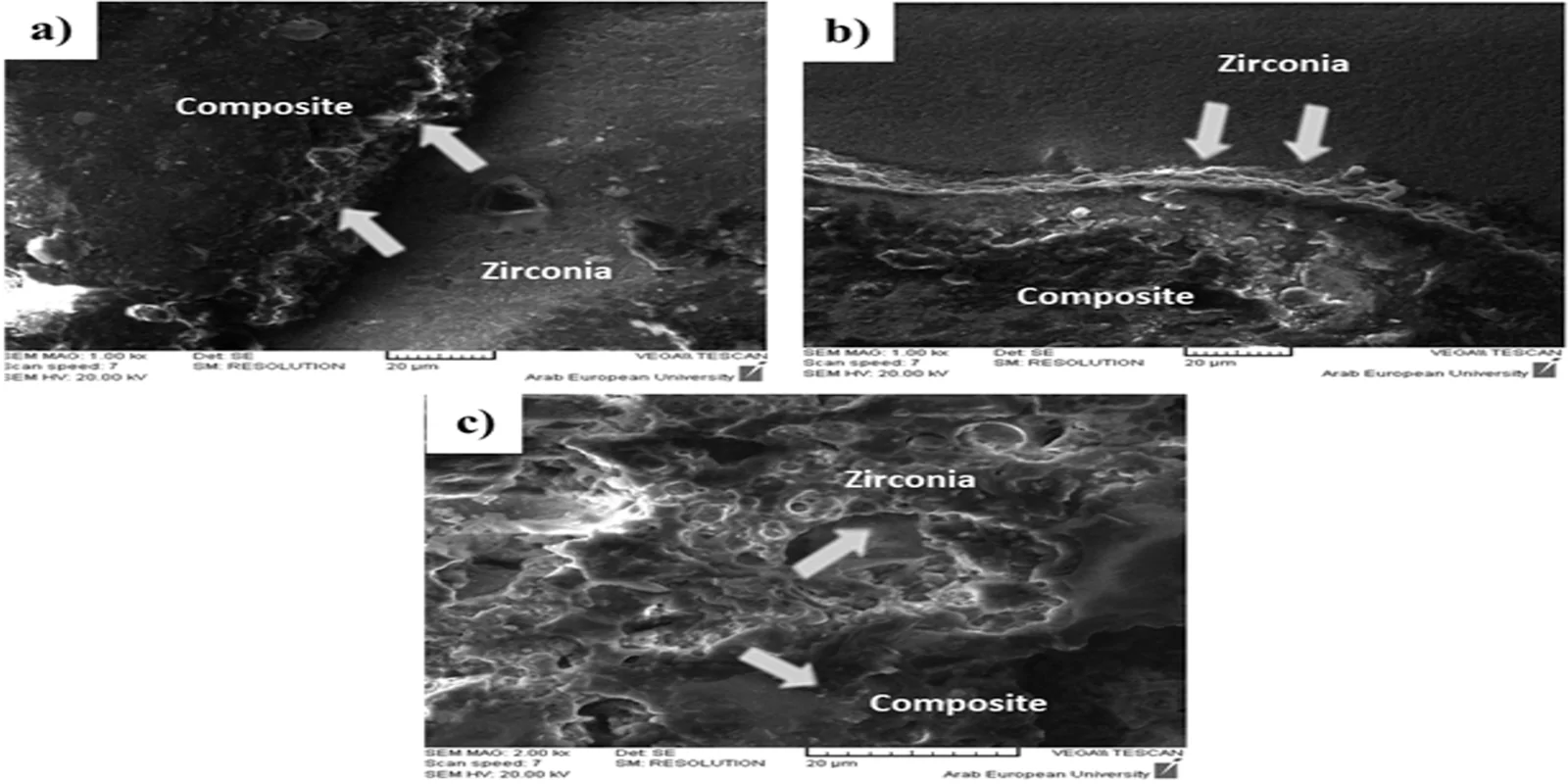
SEM Image (1000X) of the zirconia/resin interface of samples treated with 50 μm particles (a), showing lack of surface irregularity and retention properties and this is similar to CoJet group (b), while SEM Image (2000X) of the zirconia/resin interface of SIE-treated samples demonstrates resin infiltration of the 3D nanopores (c).

SEM micrographs revealed distinct morphological differences at the zirconia–resin interfaces across the surface treatment group. In the sandblasted specimens (a), which used 50 μm Al _2_O _3_ particles, the interface appeared relatively smooth, exhibiting few micro-retentive features and limited mechanical interlocking. A similar pattern was observed in the CoJet group (b), where only surface irregularities were present, offering minimal enhancement in surface roughness and resin penetration. In contrast, the samples treated with the SIE technique (c) exhibited longer micro- and nanoscale porosity on the zirconia surface, with clear resin infiltration within the three-dimensional interconnected nanopores. This indicated robust micromechanical interlocking and close interfacial contact. These observations underscore the superior adhesive performance of the SIE technique, as increased surface roughness facilitates both mechanical retention and chemical interaction with MDP-containing resin cements ( Figure 5).

### 2- Statistical results

Surface roughness: Among the study groups, Group 3 exhibited the highest surface roughness with a mean value of 1.53 μm, followed by Group 1 at 0.24 μm, and Group 2, which recorded the lowest value at 0.16 μm. Consistency was observed between the surface roughness values obtained using the TR200 and AFM methods ( Table 1). An ANOVA test was conducted to compare surface roughness among the groups (F = 496.096, p < 0.001). No statistically significant difference between Group 1 and Group 2 (p > 0.05). However, Group 3 differed significantly from both Group 1 and Group 2 (p < 0.001), as detailed in Table 2. Tukey’s post-hoc comparisons further confirmed significant differences in the average surface roughness across the groups ( Table 2).

**Table 1.** Mean values (SD) of Surface roughness, Shear bond strength recorded in each study group.

Group	N	Surface roughness by TR200, μm Mean (SD)	Surface roughness by AFM, μm Mean (SD)	Shear bond strength, Mpa Mean (SD)
Sandblasted	12	0.24 ± 0.03	0.076 ± 0.11	7.32 ± 0.57
CoJet	12	0.16 ± 0.01	0.07 ± 0.18	6.89 ± 0.50
SIE	12	1.53 ± 0.20	6.73 ± 0.23	19.36 ± 0.33

**Table 2.** Tukey’s post-hoc comparisons between each of the two groups to find the significant differences in the averages of surface roughness between the study groups.

Group I	Group J	Average difference I-J	Standard error	probability value
Sandblasted	CoJet	0.0778	0.0487	0.261
	SIE	−1.289	0.0487	**0.001>** *
CoJet	SIE	−1.367	0.0487	**0.001>** *

Tukey’s Post-hoc,

* Statistically significant.

Shear bond strength: In this study, SBS was highest for Group 3, at 19.36 MPa, followed by Group 1 at 7.32 MPa and Group 2 at 6.89 MPa ( Table 1). The ANOVA test revealed statistically significant differences among the groups (F = 2344, p < 0.001). Specifically, significant differences were observed between Group 3 and Group 2 (p < 0.001) and between Group 3 and Group 1 (p < 0.001). However, no statistically significant difference was observed between Group 2 and Group 1 (p > 0.05). Tukey’s post-hoc comparisons were conducted to identify significant differences in the average shear bond strength between the study groups ( Table 3).

**Table 3.** Tukey’s post-hoc comparisons between each two groups to find the significant differences in the averages of shear bond strength between the study groups.

Group I	Group J	Average difference I-J	Standard error	probability value
Sandblasted	CoJet	0.950	0.127	0.236
	SIE	−7.308	0.127	**0.001>** *
CoJet	SIE	−7.981	0.127	**0.001>** *

Tukey’s Post-hoc,

* Statistically significant.

**Failure modes:** The results indicated statistically significant differences in the distribution of failure modes after surface treatments, as assessed by the chi-square test. In the sandblasted group, adhesive failures were predominant, occurring in 83.3% of specimens, indicating the weakest interfacial bonding. Similarly, The CoJet group exhibited an adhesive failure rate of 66.7%. In contrast, the SIE group demonstrated a substantial improvement in bonding, with no instances of adhesive failure. Instead, this group exhibited failure modes of 75% cohesive and 25% mixed, reflecting a significantly stronger, more durable bond. Overall, these findings highlight the superior bonding performance achieved with the SIE surface treatment method. ( Table 4).

**Table 4.** Frequencies (N), percentages (%) and results of Chi-square test for comparison between failure modes after different surface treatments.

Surface treatment	Sandblasted		CoJet		SIE		Chi-value	p-value
Failure mode	N	%	N	%	N	%	28.000	**0.001>** *
Adhesive	10	83.3	8	66.7	0	0		
Mixed	2	16.7	4	33.3	3	25		
Cohesive	0	0	0	0	9	75		

* Statistically significant at p < 0.05.

## Discussion

Ceramic restorations are extensively used due to their superior aesthetic results and biocompatibility. ^5, 13^ Among these materials yttria-stabilized tetragonal zirconia polycrystal (Y-TZP) has garnered significant attention for its outstanding mechanical properties. ^14, 15^However, a major clinical challenge is achieving a durable bond to zirconia. This bond is critical to prevent fractures and debonding during cementation, ensuring the durability and longevity of the restoration. ^16^ Numerous surface treatments have been developed to enhance the bonding between resin and zirconia. ^4, 17^

A common protocol for bonding zirconia involves airborne-particle abrasion (sandblasting), followed by the application of a monomer known as MDP to achieve chemical bonding. ^4, 8, 18^ However, this approach can induce a tetragonal-to-monoclinic phase transformations, which may compromise the material’s integrity. ^12, 19^ An alternative method is the tribochemical silica coating technique, ^8, 20^ which involves depositing a silica layer onto the zirconia surface. This coating facilitates silane coupling and enhances chemical bonding with resin cements. ^20^ Nonetheless, air abrasion may still introduce surface flaws that may compromise strength. ^8^ More recently, SIE has been proposed as a way to improve the adhesive potential of zirconia, ^8^ and initial results have been promising. Despite these advancements, the wide variety of materials and techniques available means that no definitive gold standard for zirconia bonding has yet been established. ^3^

This study evaluated the effectiveness of three surface treatment techniques CoJet, SIE, and conventional methods. It also investigated the influence of MDP-containing resin adhesives on the long-term bond strength of zirconia ceramics after undergoing artificial aging. The shear bond strength test was employed to assess the effectiveness of the bond strength of zirconia. Additionally, the effects of different surface treatments on the microstructure of zirconia specimens were analyzed using SEM, Energy Dispersive X-ray Spectroscopy (EDX), and AFM topographic analyses. The failure modes were further examined using SEM.

Conventional sandblasting enhances bond strength primarily by generating micro-rough surfaces for mechanical interlocking. However, this process can induce microcracks and phase transformations, which may compromise the long-term structural integrity and fracture toughness of ceramics. Moreover, sandblasting does not inherently improve surface wettability or chemical reactivity. ^8, 21^ In contrast, TSC employs a dual mechanism, combining micromechanical retention with the potential for chemical bonding via a deposited silica layer. Despite these advantages, the efficacy of TSC can be limited by the inconsistent adherence and non-uniformity of the silica layer, which can affect the durability of the bond. ^22^ The SIE technique stands out for its superior performance, as it marks a significant departure from traditional abrasive methods like sandblasting and TSC. ^11^ SIE is a chemical process that selectively generates a sophisticated three-dimensional nano-porous network at the grain boundaries of zirconia without causing phase transformation or surface defects. The primary bonding mechanism here is nano-mechanical interlocking; the resin cement penetrates deeply and polymerizes within this intricate subsurface porosity, resulting in a highly integrated and robust bond. ^23^ Crucially, SIE achieves this exceptional adhesion without applying damaging mechanical stresses, which helps preserve the inherent mechanical properties of zirconia. ^24^

The results of this study align with findings from previous research, ^23– 25^ which demonstrated that SIE demonstrated superior bond strength durability compared to other groups. However, in this present study, SIE did not achieve a higher surface roughness than the sandblasting group.

This discrepancy may be due to a difference in the mixture composition, which could have hindered its effectiveness in penetrating the surface. ^23^ Furthermore, Saade et al. ^26^ examined the impact of various surface treatment combinations on the adhesion of resin composites to zirconia. They reported that different treatment protocols did not produce statistically significant differences in bond strength values. Supporting this, another study found no statistically significant difference in bond strength between the sandblasting and silica coating groups. Moreover, the study highlighted that the manufacturer’s recommended blasting pressure of 2.8 bar was insufficient to complete the tribochemical process. ^22^ Conversely, using a higher blasting pressure poses a risk of damaging the zirconia surface by inducing phase transformation. Following a different protocol for CoJet and sandblasting applications can lead to varying outcomes. ^15^

In adhesive dentistry, the mode of failure serves as a critical indicator of bond strength. Adhesive failures, occurring at the interface, are the least desirable outcome. In contrast, Cohesive failures, which happen within the material itself, are more favorable as they imply that the bond is stronger than the material. Combined failures are considered an acceptable mix. A direct correlation exists between higher shear bond strength and an increased percentage of cohesive and mixed failures. Therefore, a high proportion of cohesive and mixed failures indicates a strong and clinically successful bond.

In this study, the failure rate of sandblasting group was 83.3% adhesive and 16.7% mixed, this may be attributed to weak bonding strength. The CoJet group recorded 66.7% adhesive, 33.3% mixed failures. Although the failure cohesive in the group SIE was 75%, the mixed failure rate was 25%, indicating a good bonding strength.

There was no significant difference in bond strength between the sandblasting and CoJet groups. However, a mixed failure pattern was noted in the CoJet group, which could be explained by the fact that many silica particles did not fuse effectively and remained as loosely attached particles. ^22^ As a result, this hindered adequate cement bonding. The mixed failure pattern observed in the sandblasting group can be attributed to the presence of larger pits.

The SEM evaluation of the samples, as illustrated in Figure 2, revealed that the control group had a rough surface with larger pits and sharp edges. However, this mild roughness was insufficient to produce a retentive surface compared to the other surface treatments, while In the CoJet group, clusters of loosely covered silica particles were observed on the surface, forming chemically reactive “islets.” These islets may have chemically modified the zirconia surface, enabling better reaction with the primers. Additionally, Microcracks, some roughness, and porosities were noted, with the microcracks acting as points of stress concentration.

In contrast, the SIE-treated specimens exhibited a compact glassy layer with rounded fused beads prior to etching. After etching, a distinct honeycomb-like nanoporous structure was observed, indicating the successful infiltration and selective dissolution of the glassy phase. These morphological changes directly explain the superior micromechanical and chemical bonding performance of the SIE group. The SEM findings provide visual confirmation that the physical characteristics of the treated surfaces align with their measured bond-strength values. The limited microretentive surface of sandblasting and the irregular silica deposition in the CoJet group explain their lower adhesion. Conversely, the well-defined nanoporous architecture of the SIE group ensures deep resin penetration and superior interfacial integration.

This study was conducted in a laboratory setting that simulated the oral environment through thermocycling. However, one of the main limitations is the absence of dynamic loading and saliva simulation. This limitation may restrict the direct applicability of the in vitro findings to real clinical conditions.

## Conclusions

In this in-vitro study, SIE demonstrated greater effectiveness as a surface modification technique for translucent zirconia when compared to conventional air-abrasion and CoJet systems. The specimens treated with SIE exhibited the highest shear bond strength values and predominantly cohesive failure modes. This confirms the formation of a strong and durable interface between zirconia and resin. SEM analysis supported these findings, revealing distinctive honeycomb-like nanoporous structure in the SIE group This structure facilitated a high level of resin infiltration. In contrast, the sandblasted and CoJet surfaces displayed irregular grooves and loosely attached silica clusters, resulting in less effective retention. The improved adhesion observed with SIE is attributed to its ability to generate a three-dimensional nanoporous network. This network enhances micromechanical interlocking and resin penetration while avoiding damage to the surface or phase transformation. In comparison, traditional sandblasting and tribochemical silica coating techniques offer limited improvements in bond performance and may introduce microdefects that compromise long-term stability. To further substantiate these results under intraoral conditions, future studies should incorporate dynamic mechanical loading and clinical simulation. This would also help refine the SIE protocol for clinical application.

## Ethics

Ethical approval was obtained from the Damascus University Ethics Committee.

## Data Availability

All datasets required to reproduce the results reported in this article are included. Zenodo: Selective Infiltration Etching as a surface modification for high-translucency zirconia: Comparative analysis of roughness, composition, and bond strength. https://doi.org/10.5281/zenodo.18213115 ^27^ The project contains the following underlying data:
• Surface roughness values for all samples using AFM in xlsx 1 • Surface roughness values for all samples using TR200 in xlsx 1 • Shear bond strength values for all samples in xlsx 1 • failure modes for all samples in xlsx 2 Repository name: Selective Infiltration Etching as a surface modification for high-translucency zirconia: Comparative analysis of roughness, composition, and bond strength. https://doi.org/10.5281/zenodo.18213115 ^27^ This project contains the following extended data:
-AFM_images.tif (atomic microscope images of the zirconia surface after treatments) in figure 1
SEM_surface_images.tif (scanning electron microscope images of the zirconia surface after treatments in figure 2 EDX analysis showed considerable compositional variations in the treated zirconia surfaces in figure 3. -SEM_failure_modes.tif (scanning electron microscope images for three failure modes) in figure 4,5. Supplementary_Table1.xlsx (Summary statistics): • Mean and standard deviation values of surface roughness formed by TR200 and AFM • Mean and standard deviation values for shear bond strength as reported in Table 1 • Tukey’s post-hoc comparisons between groups to identify significant differences in surface roughness (Table 2) • Tukey’s post-hoc comparisons between groups to identify significant differences in shear bond strength (Table 3) • Frequencies (N), percentages (%), and Chi-square test results for comparison between failure modes after different surface treatments (Table 4) Data are available under the terms of the Creative Commons Attribution 4.0 International license (CC-BY 4.0).
