# Supplementary material for: Selective Infiltration Etching as a surface modification for high-translucency zirconia: Comparative analysis of roughness, composition, and bond strength
Source: F1000Res. 2026 Jul 8;15:562. Originally published 2026 Apr 18. [Version 2] doi: 10.12688/f1000research.176295.2 (PMC13554812; doi:10.12688/f1000research.176295.2)
Supplement: Peer review history

# Reviewer report: Reviewer response for version 2

**DOI:** 10.5256/f1000research.204583.r501032

**Published:** 2026-07-30

**Authors:** Dina Mohamed Nasr

**Affiliations:** 1 Alexandria University, Alexandria, Egypt

High translucency zirconia is a highly promising material for dental prosthetics due to its biocompatibility, mechanical strength and better esthetics, therefore this study focused on zirconia bonding using previously accepted methods for conventional tetragonal zirconia surface treatment. In this study , the effect of surface treatment on surface roughness, surface properties, and bond strength were examined to determine which surface treatment would be recommended to enhance bonding to high translucency zirconia.  This work is reproducible and methods of examination are well presented, however, it would be more beneficial if Figure 1 can be presented in a better resolution  and Figure 2 is enhanced regarding its quality and better showing of image data below each image.

 Regarding my clinical point of view, I would recommend testing of restoration fit and marginal and internal adaptation after selective infiltration etching, as I think that this method and coating the fitting surface of restoration with a laboratory-prepared paste with subsequent preparation techniques, would interfere with restoration seating clinically.

Is the work clearly and accurately presented and does it cite the current literature?

Yes

If applicable, is the statistical analysis and its interpretation appropriate?

Yes

Are all the source data underlying the results available to ensure full reproducibility?

Partly

Is the study design appropriate and is the work technically sound?

Yes

Are the conclusions drawn adequately supported by the results?

Yes

Are sufficient details of methods and analysis provided to allow replication by others?

Yes

Reviewer Expertise:

adhesive dentistry, prosthodontics, dental material science

I confirm that I have read this submission and believe that I have an appropriate level of expertise to confirm that it is of an acceptable scientific standard.

# Reviewer report: Reviewer response for version 2

**DOI:** 10.5256/f1000research.204583.r499685

**Published:** 2026-07-20

**Authors:** Rafat Sasany

**Affiliations:** 1 Biruni university, Samsun, Turkey

Have no comments, it's really well done.

Is the work clearly and accurately presented and does it cite the current literature?

Partly

If applicable, is the statistical analysis and its interpretation appropriate?

Yes

Are all the source data underlying the results available to ensure full reproducibility?

Partly

Is the study design appropriate and is the work technically sound?

Partly

Are the conclusions drawn adequately supported by the results?

Yes

Are sufficient details of methods and analysis provided to allow replication by others?

Yes

Reviewer Expertise:

Prosthodontics, Adheziev, Digital dentistry, dental materials

I confirm that I have read this submission and believe that I have an appropriate level of expertise to confirm that it is of an acceptable scientific standard.

# Reviewer report: Reviewer response for version 1

**DOI:** 10.5256/f1000research.194339.r477634

**Published:** 2026-05-09

**Authors:** Awiruth Klaisiri

**Affiliations:** 1 Thammasat University, Pathum Thani, Thailand

1. How did you check the defects in the specimen? Please clarify.
2. How did you control the bonding area? Please clarify.
3. How did you control the film thickness of resin cement? Please clarify.
4. Please clarify and fill in deep detail for this sentence: "Cementation was performed under a constant load using a standardized metal device."
5. Please clarify the blade in the SBS test (notch-edge or knife blade) and the reason you use the blade.
6. Please clarify the details of SEM.
7. Please answer the hypothesis in the discussion section.
8. Please add the clinical application.
9. Please rewrite the conclusion.

Is the work clearly and accurately presented and does it cite the current literature?

Yes

If applicable, is the statistical analysis and its interpretation appropriate?

Yes

Are all the source data underlying the results available to ensure full reproducibility?

Yes

Is the study design appropriate and is the work technically sound?

Partly

Are the conclusions drawn adequately supported by the results?

Partly

Are sufficient details of methods and analysis provided to allow replication by others?

Partly

Reviewer Expertise:

Bond strength, Ceramic, Zirconia, Adhesive

I confirm that I have read this submission and believe that I have an appropriate level of expertise to confirm that it is of an acceptable scientific standard, however I have significant reservations, as outlined above.

# Response

**Published:** 2026-06-21

**Authors:** ibrahim Alghoraibi

**Affiliations:** Fixed Prosthodontics, Damascus University, Damascus, Damascus Governorate, Syria

Thank you for the valuable comments regarding our manuscript. We have taken all suggestions into consideration and updated the paper to reflect these improvements. Please find our detailed responses to each point below

1. How did you check the defects in the specimen? Please clarify.

**Response: **The surfaces of all samples from each group were examined after separation by a trained assessor who was unaware of the sample groups, using an SKT 41322 MEIJI TECHNO dissecting microscope. The failure modes were classified according to ISO 10365:2022 E.

 The failure percentage was determined as follows:

 A failure was classified as adhesive when approximately 25% of the cement remained on the fractured surface, cohesive when approximately 75% remained, and mixed when the remaining cement percentage fell between these two values.

  

1. How did you control the bonding area? Please clarify.

**Response: **Circular adhesive sheets were fabricated and precisely punched using a specialized tool to create a defined 3 mm diameter hole. The sheets were then applied to the zirconia surfaces to standardize the working area.

  

1. How did you control the film thickness of resin cement? Please clarify.

**Response: **A constant pressure of 5 kg was applied to the samples using a pressure-standardizing device in order to ensure uniform cement thickness and standardize the testing conditions. The device was located in the Department of Fixed Prosthodontics, Faculty of Dentistry, University of Damascus.

  

1. Please clarify and fill in deep detail for this sentence: "Cementation was performed under a constant load using a standardized metal device."

**Response: **The term "constant load" refers to the pressure-standardizing device mentioned in the previous section.

  

1. Please clarify the blade in the SBS test (notch-edge or knife blade) and the reason you use the blade.

**Response: **The blade used in the shear test was a chisel-edge blade, as the chisel method is considered more practical, faster, and easier to handle during testing. This method is recommended by ISO/TS 11405:2003 and has been adopted in numerous previous studies (Baiomy et al 2023) and these are references (1) in the article.

1. Please clarify the details of SEM.

**Response: **The aluminum holder of the electron microscope was mounted on the plastic base, and double-sided carbon adhesive tape was applied to the holder. The sample was then placed with the zirconia surface facing upward. The aluminum holder with the sample was transferred to the examination stage using tweezers, and the microscope chamber was closed.

1. Please answer the hypothesis in the discussion section.

**Response: **A paragraph has been added to the Discussion section addressing whether the results support the initial hypothesis.

 The study hypothesis was partially rejected. While significant differences were observed between Group 3 and the other two groups (Groups 1 and 2) regarding both surface roughness and shear bond strength (p < 0.001), no statistically significant differences were found between Group 1 and Group 2 (p > 0.05). Consequently, the hypothesis that all groups would differ significantly from one another was only partially supported.

  

1. Please add the clinical application.

**Response: **Clinical Application

 A paragraph has been added to emphasize the clinical relevance of the findings, highlighting their importance in improving bonding between ceramic and zirconia in dental applications.

 "Heat-induced maturation and selective infiltration etching produced a reliable and profound zirconia-resin bond strength resistant to artificial aging, thereby enhancing the long-term clinical longevity of the restorations. Furthermore, these findings support the clinical feasibility of using translucent zirconia veneers as a durable and aesthetic restorative option."

  

1. Please rewrite the conclusion.

**Response: **The Conclusion has been rephrased to clearly summarize the main findings and provide practical recommendations.

 Conclusions

 In this in-vitro study, SIE proved to be more effective than conventional air-abrasion and CoJet systems for modifying translucent zirconia surfaces. SIE-treated specimens showed the highest shear bond strength and mainly cohesive failures, indicating a strong and durable zirconia-resin interface. SEM analysis revealed a honeycomb-like nanoporous structure that enhanced resin infiltration, whereas sandblasted and CoJet surfaces exhibited irregular grooves and loosely attached silica clusters, resulting in lower retention. The superior adhesion with SIE is attributed to its three-dimensional nanoporous network, which improves micromechanical interlocking without causing surface damage. Further studies simulating intraoral conditions are recommended to validate these findings and optimize the clinical SIE protocol.

 We hope that these revisions address all concerns raised, and we sincerely appreciate the reviewer’s time and constructive suggestions.

# Reviewer report: Reviewer response for version 1

**DOI:** 10.5256/f1000research.194339.r480930

**Published:** 2026-05-05

**Authors:** Rafat Sasany

**Affiliations:** 1 Biruni university, Samsun, Turkey

1. The null hypothesis is incomplete because it mentions only roughness, morphology, and composition, but the main outcome includes shear bond strength. The hypothesis should also include bond strength.
2. Although 36 specimens were used, only 12 specimens per group were tested, and SEM/EDX evaluation was performed on only 2 specimens per group. This is limited for strong microstructural interpretation.
3. The study used 5000 thermocycles, but no mechanical fatigue loading or saliva simulation was included. Therefore, the results may not fully represent clinical durability.
4. The ANOVA F value for shear bond strength is reported as “F = 2344,” which seems unusually high and may be a reporting or decimal error. Exact p values should be reported instead of “0.001>”.
5. The results state that SIE had the highest surface roughness, but the discussion says “SIE did not achieve a higher surface roughness than the sandblasting group.” This contradiction must be corrected.
6. The cementation area, cylinder diameter, bonding jig, applied load value, and failure-area calculation are not sufficiently described. This limits reproducibility.
7. The exact brand, type, yttria content, and translucency class of the zirconia material should be clearly reported.
8. The conclusion claims superior durability, but only short-term thermocycling was performed. The authors should avoid strong clinical claims without fatigue testing or long-term aging.
9. The authors are encouraged to include and discuss the following relevant study (DOI: 10.1080/01694243.2020.1869398), which is closely related to zirconia surface treatments and bonding performance, in order to strengthen the scientific background and contextualize their findings.

Is the work clearly and accurately presented and does it cite the current literature?

Partly

If applicable, is the statistical analysis and its interpretation appropriate?

Yes

Are all the source data underlying the results available to ensure full reproducibility?

Partly

Is the study design appropriate and is the work technically sound?

Partly

Are the conclusions drawn adequately supported by the results?

Yes

Are sufficient details of methods and analysis provided to allow replication by others?

Yes

Reviewer Expertise:

Prosthodontics, Adheziev, Digital dentistry, dental materials

I confirm that I have read this submission and believe that I have an appropriate level of expertise to confirm that it is of an acceptable scientific standard, however I have significant reservations, as outlined above.

# Response

**Published:** 2026-06-21

**Authors:** ibrahim Alghoraibi

**Affiliations:** Fixed Prosthodontics, Damascus University, Damascus, Damascus Governorate, Syria

We would like to sincerely thank the reviewer for the valuable feedback and constructive comments. We have carefully addressed each point raised, and the manuscript has been revised accordingly. Below is a detailed response to each comment:

 1. The null hypothesis is incomplete because it mentions only roughness, morphology, and composition, but the main outcome includes shear bond strength. The hypothesis should also include bond strength.

**Response: **We acknowledge that the original null hypothesis mentioned only roughness, morphology, and composition.

 We have revised it to include shear bond strength as a primary outcome, ensuring alignment with the main results.

 The null hypothesis of this study is that Selective Infiltration Etching (SIE) does not significantly affect surface roughness, morphology, and composition , nor does it significantly influence the shear bond strength (SBS) of translucent zirconia when compared to traditional surface treatment methods.

 2. Although 36 specimens were used, only 12 specimens per group were tested, and SEM/EDX evaluation was performed on only 2 specimens per group. This is limited for strong microstructural interpretation.

**Response: **The study included a total of 36 samples, distributed equally among three subgroups (12 samples per subgroup). Consequently, all 36 samples were subjected to shear bond strength testing. However, EDX analysis was limited to two representative samples because of the high cost associated with this technique.

 3. The study used 5000 thermocycles, but no mechanical fatigue loading or saliva simulation was included. Therefore, the results may not fully represent clinical durability.

**Response: ** The reviewer correctly noted that 5000 thermal cycles alone do not fully simulate clinical conditions.

 We have clarified this limitation and stated that long-term mechanical fatigue and saliva simulation were not included, which may affect clinical extrapolation.

 4. The ANOVA F value for shear bond strength is reported as “F = 2344,” which seems unusually high and may be a reporting or decimal error. Exact p values should be reported instead of “0.001>”.

**Response: **We appreciate the reviewer’s comment. We confirm that the reported F value (F = 2344) for shear bond strength is correct. The unusually high F value reflects the substantial differences observed between the subgroup means, which indicates a strong effect of the tested variable on shear bond strength. We have double-checked the calculations to ensure accuracy. Regarding the p value, we have provided the exact value in the revised manuscript for clarity, and was 0.001.

 5. The results state that SIE had the highest surface roughness, but the discussion says “SIE did not achieve a higher surface roughness than the sandblasting group.” This contradiction must be corrected.

**Response: **Thank you for highlighting this point. The statement in the Discussion section refers to the findings of a previous study (Salem et al 2016), and it's reference (23) and not to the results of the current investigation. Our results clearly showed that SIE exhibited the highest surface roughness. The text has been revised to clarify this distinction and prevent any potential confusion.

 6. The cementation area, cylinder diameter, bonding jig, applied load value, and failure-area calculation are not sufficiently described. This limits reproducibility.

**Response:** In the present study, the bonding interface was circular with a cylinder diameter of 3.0 mm, yielding a total cementation area of 7.07 mm². To ensure a uniform cement thickness and standardize the testing conditions, a specialized pressure-standardizing device was utilized. This device, located in the Department of Fixed Prosthodontics, Faculty of Dentistry, University of Damascus, maintained a constant static load of 5.0 kg on the samples during the setting phase. The maximum load to failure was recorded, and the SBS was calculated as follows: SBS (MPa) = load (N)/area (mm²).

 7. The exact brand, type, yttria content, and translucency class of the zirconia material should be clearly reported.

**Response: **The zirconia used in this study was Cube X² (Dental Direkt, Germany), a high-translucency zirconia with a yttria content of [5 mol% Y₂O₃] and classified as [5Y-TZP]. These details have been added to the Materials and Methods section to ensure complete characterization of the material and facilitate reproducibility.

 8. The conclusion claims superior durability, but only short-term thermocycling was performed. The authors should avoid strong clinical claims without fatigue testing or long-term aging.

**Response: **We agree that the term “superior durability” may be considered strong given that only short-term thermocycling was performed without long-term aging or fatigue testing. Therefore, the conclusion has been revised to avoid overgeneralization and to better reflect the in vitro nature and limitations of the study. The revised conclusion now emphasizes the short-term performance of the tested materials rather than long-term clinical durability.

 9. The authors are encouraged to include and discuss the following relevant study (DOI: 10.1080/01694243.2020.1869398), which is closely related to zirconia surface treatments and bonding performance, in order to strengthen the scientific background and contextualize their findings.

**Response: **The recommended study (DOI: 10.1080/01694243.2020.1869398) has been reviewed and cited in the revised manuscript. It has been briefly incorporated into the Introduction and sections to strengthen the scientific background and contextualize our findings on zirconia surface treatments and bonding performance.
